# Constrained trajectory optimization and force control for UAVs with universal jamming grippers

**DOI:** 10.1038/s41598-024-62416-1

**Published:** 2024-05-25

**Authors:** Paul Kremer, Hamed Rahimi Nohooji, Holger Voos

**Affiliations:** 1 Automation & Robotics Research Group, Interdisciplinary Centre for Security, Reliability and Trust (SnT), University of Luxembourg, Esch-sur-Alzette, Luxembourg; 2 Faculty of Science, Technology and Medicine (FSTM), University of Luxembourg, Esch-sur-Alzette, Luxembourg

**Keywords:** Computational science, Aerospace engineering

## Abstract

This study presents a novel framework that integrates the universal jamming gripper (UG) with unmanned aerial vehicles (UAVs) to enable automated grasping with no human operator in the loop. Grounded in the principles of granular jamming, the UG exhibits remarkable adaptability and proficiency, navigating the complexities of soft aerial grasping with enhanced robustness and versatility. Central to this integration is a uniquely formulated constrained trajectory optimization using model predictive control, coupled with a robust force control strategy, increasing the level of automation and operational reliability in aerial grasping. This control structure, while simple, is a powerful tool for various applications, ranging from material handling to disaster response, and marks an advancement toward genuine autonomy in aerial manipulation tasks. The key contribution of this research is the combination of a UG with a suitable control strategy, that can be kept relatively straightforward thanks to the mechanical intelligence built into the UG. The algorithm is validated through numerical simulations and virtual experiments.

## Introduction

The evolution of UAVs over the past decade has significantly transformed the landscape of numerous scientific and industrial domains^1–4^. Initially envisioned as aerial sensors, UAVs demonstrated unparalleled utility in surveillance^5,6^, forest fire monitoring^7^, building inspection^8,9^, forestry^10^ and terrain mapping^11^ due to their agility and accessibility to challenging environments^12,13^. However, despite their remarkable success in sensing and monitoring, UAVs initially struggled in scenarios that demanded physical interaction with the environment.

Emerging as a solution, the field of aerial manipulation sought to equip UAVs with robotic manipulators^14–16^ and claw-like grippers^17–19^. This marked the beginning of a new era where UAVs could engage in tasks requiring direct physical contact^20^. However, these traditional claw-like rigid grippers required precise alignment and positioning relative to the target object^21^. Even minor deviations could prevent successful grasping, making them susceptible to inaccuracies and necessitating exhaustive grasp analysis.

Soft aerial grasping emerged as an evolution to address the challenges posed by traditional grasping mechanisms. Soft grippers, with their adaptability and compliance, can seamlessly fit various object geometries, significantly minimizing the need for detailed grasp analysis^22–26^. Inherent to soft robotics is their lightness and versatility thanks to the passive intelligence baked into their mechanical structure. Not only does this reduce the mechanical impact during interactions but it also makes the grippers particularly suitable for aerial applications. The inherent compliance of soft robotics presents an innovative solution to the difficulties related to tolerance in object grasping^25^. Their ability to passively conform to objects leverages the concept of morphological computation, where the gripper’s passive mechanical structures supplement the role of active controls^27,28^.

A particular type of soft gripper was introduced in^29^, called the universal jamming gripper (UG). The UG is based on the principle of granular jamming where certain materials transition from a fluid- to a solid-like state when subjected to external pressure^30^. This unique mechanism allows particles to flow and take arbitrary shapes, then solidify in place under external pressure^31^. The UG’s lightweight composition features a membrane filled with such a material that hardens under vacuum, establishing a firm grasp on the englobed object via a combination of suction, physical interlocking, and friction. Unlike many other soft grippers, the UG is specially crafted to engage with a wide array of object geometries^32–34^ and delicate manipulations^35^. Its symmetric design, omnidirectional grasping capabilities, and passive compliance sidestep some of the complexities inherent to traditional grippers, giving room to simpler control strategies.

Guidance and control strategies are the foundational pillars in aerial manipulation literature. Typically, trajectory generation and low-level tracking control are used to meet the mission requirements^36,37^. The complexity of the tracking controller generally depends on the type of aerial manipulator. AMs equipped with heavy serial link manipulators require their dynamics to be taken into account^38^. In contrast, simple claw-like grippers can often be treated as an unknown disturbance, effectively handled by common model-free robust control schemes such as PID or SMC. Trajectory generation often distinguishes between global and local trajectories, where global trajectories require path planning utilizing some form of map representation^37^. Local, short-term, trajectory generation is frequently formulated as an optimization problem, specifically adapted to the nature of the mission. For instance, B-spline and sequential quadratic programming were used in^39^ to generate minimum-time trajectories. Real-time minimal jerk trajectories for aerial perching were shown in^40^, while multi-objective optimization-based trajectory planning was demonstrated in^41^. Dynamic artificial potential fields were used to follow a moving ground target in^42^. Lastly, stochastic model predictive control (MPC) and dynamic movement primitives were applied in^43^ and^44^.

MPC is a control strategy that leverages online optimization techniques to compute optimal control inputs over a receding horizon according to a given objective function. It was used with great success in many domains^45,46^ thanks to its ability to work with non-linear system models and its ability to respect arbitrary constraints. A common problem of this technique is the computational burden related to the optimization step, which was especially problematic for systems exhibiting fast dynamics with limited compute resources (e.g., UAVs). However, in recent years, embedded systems have become more powerful, and optimization algorithms have become more efficient^47^. In UAV trajectory generation, MPC can fully exploit the system’s dynamics while respecting constraints imposed by the environment and the manipulation task. Its ability to handle multiple (potentially conflicting) objectives and  its robustness to dynamical uncertainties thanks to the receding horizon approach, makes it a prime candidate for local trajectory generation.

Our previous work^34^ experimentally validated the UG’s grasping capabilities on a real UAV in an *open-loop* setup, highlighting the benefits of an automated approach. The reliance on a human in the loop inherently limits the scalability and adaptability of UAV operations in grasping tasks. Addressing this shortcoming, this study melds the inherent passive intelligence of the gripper with a simple, robust control scheme, marking a significant leap towards fully automated grasping.

The main objective of this paper is to introduce a novel control architecture for aerial grasping with soft jamming grippers, addressing the prevalent challenges in UAV deployments for material handling and grasping tasks. This initiative is realized by customizing a soft universal jamming gripper for aerial grasping and developing a model predictive controller to constrain the UAV’s motion while enhancing operational autonomy and safety. The contributions of this research encompass:

- Formulation and implementation of a constrained path planning algorithm, specifically designed for navigating UAVs in complex aerial environments, utilizing model predictive control (MPC) adapted to the UG.
- Integration and application of a force control strategy, ensuring both efficacy and robustness in grasping operations.
- Presentation and validation of a scenario that showcases the framework’s efficacy, contributing to elevating the level of autonomy in aerial grasping tasks, verified through simulations and virtual experiments.

The salient features of this framework are its simple structure, straightforward architecture, and the synergetic effects between the control system and the passive intelligence of the UG to solve an otherwise complex problem.

The paper is structured as follows: We begin by articulating the problem statement and outlining the necessary technical background, followed by a deep dive into MPC where we elaborate on its formulation and adaptations tailored for the proposed grasping mechanism. Next, we introduce the force control strategy that dictates the gripping actions, ensuring both secure and reliable grasping. After this, we present the numerical simulation process, followed by a description of the virtual experiments conducted to validate our framework, highlighting the simulations and results that underscore the effectiveness of our approach. We then discuss and interpret the findings, explore their implications, and suggest future research directions. Finally, we conclude by summarizing the key takeaways of our research.

## Problem statement and preliminaries

### Problem statement

The main objective of this research is to enhance autonomy in aerial grasping tasks involving a UAV equipped with a UG. Achieving this autonomy necessitates overcoming several challenges:

*Optimal Approach Planning.* The UAV, armed with a UG, must efficiently navigate toward the target object in cluttered and potentially changing environments. The planning algorithm should be robust, adaptable, and capable of handling real-time environmental perturbations, ensuring that the UAV can modify its trajectory dynamically to avoid obstacles and save energy.

**Figure 1:**
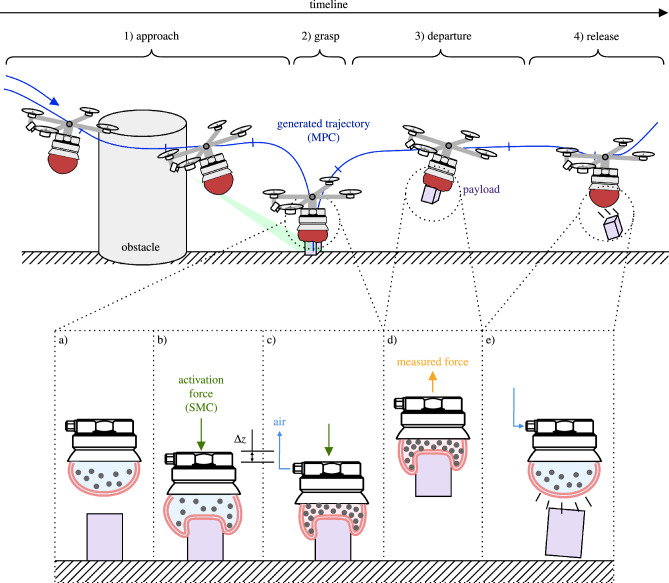
Schematics of autonomous grasping setup with a UG-equipped UAV. (1) The UAV approaches the payload avoiding all obstacles by tracking the MPC-generated trajectory. The payload’s position is roughly known, and then refined as it appears in the FoV of the camera. (2) As the gripper makes contact with the payload, the grasping procedure is started and the UAV switches into force control mode (SMC). (3) Once a reliable grasp is established, the UAV departs with the payload. (4) Upon reaching its destination, the payload is released. (**a**) The membrane is filled with air and a granular material. (**b**) An activation force pushes the membrane against the target, partially englobing the object. (**c**) Air is removed from the membrane. It shrinks and jams the particles, turning itself into a solid-like state. The contact force has to be maintained during this transition to establish a reliable grasp. (**d**) The resulting friction, geometric interlocking, and suction create a firm grasp. An integrated load cell measures the weight of the payload, used as feed-forward in the control architecture. (**e**) Pumping air into the membrane releases the object.

*Effective grasping* The grasping mechanism utilizes particle jamming and a soft membrane to establish a firm yet gentle grasp on the target object as depicted in Fig. 1a–c. The control strategies employed should control the activation force, mitigating the risks of damage during the interaction. Further, make sure that contact is maintained during the closing transition of the gripper, a necessary condition for a strong grasp. Moreover, it has to deal with the changing physical properties during the jamming transition of the gripper (from soft to rigid).

*Adaptability post-grasping* Following the capture of the payload, the UAV, in its role as an aerial manipulator (AM), must efficiently transport the payload to a designated location. This phase leverages the payload’s measured mass as a feed-forward element in the AM’s control system for rapid adjustment, Fig. 1d. This swift recalibration, essential for maintaining flight stability under increased weight, ensures safe and effective delivery.

The overarching goal is to craft a comprehensive control framework that synergizes with the innovative mechanics of the UG, resulting in a fully automated aerial grasping system. This framework should be capable of navigating the complexities of real-world environments, ensuring the seamless execution of aerial grasping tasks from approach to object retrieval and final deposition. The proposed framework and its integral components, aimed at solving the outlined problems, will be evaluated through numerical simulations and virtual experiments to validate their effectiveness and applicability in practical scenarios. The envisioned automated grasping setup is depicted in Fig. 1.

### UG modeling

This section summarizes crucial insights into the modeling of the UG, laying the foundation for the virtual experiments discussed later in the manuscript. For an in-depth exploration of the UG’s design, fabrication, integration, modeling, and experiments on a UAV, readers are directed to^34^.

Key to the UG’s capabilities is its ability to shift from a soft to a rigid state, see Fig. 1b–c. This adaptability significantly enhances the robustness of aerial manipulation tasks. Notably, the UG’s ability to adapt to various object geometries negates the need for precise control based on object orientation, thereby drastically simplifying the control complexity.

**Figure 2:**
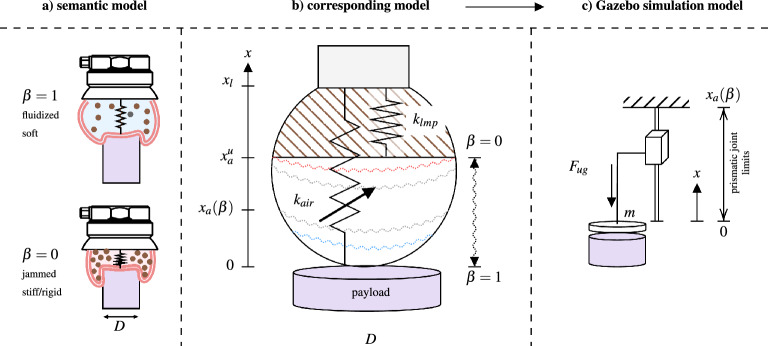
UG contact model. (**a**) Semantic model of the UG showing its soft (fluidic) state, and its rigid (closed) state. (**b**) The corresponding model delineates the membrane into two components: the air-filled elastic part and the system’s remainder (filler material and structural components), represented as compression springs $k_{air}$ and $k_{lmp}$ in a parallel configuration. (**c**) The derived simulated system encompasses a disk (body) with a mass *m*, coupled to a prismatic joint with finite, state-dependant travel, governed by the combined elastic force $F_{ug}$ that substitutes the parallel spring arrangement.

To render the UG’s behavior in robotic simulators, we propose the contact model in Fig. 2. This model melds two non-linear compression springs, $k_{lmp}$ and $k_{air}$. The former captures the lumped stiffness of the jammed filler material, while the latter encapsulates the dynamics of the air-filled membrane during contact which embodies numerous parameters, including the effective contact area and the non-linear elastic behavior of the membrane, a depiction akin to an air spring as cited in^48^. However, pinpointing a precise model proves challenging, and offers limited practical utility in this context.

We employed non-linear regression analysis to identify the interrelation between the depth of entrance *x*, the payload diameter *D*, and the resultant elastic force $F_{air}$. Concurrently, the system’s lumped elastic force is denoted as $F_{lmp}(x)$. The combined elastic force $F_{ug}$ is then derived from the equation

1 $$\begin{aligned} F_{ug} = F_{air}(h(x), D) + F_{lmp}(h(x - x_a)), \end{aligned}$$

where *h*(*y*) represents the compression-only action of the springs, defined as $h(y) = 0$ if $y < 0$, and $h(y) = y$ if $y \ge 0$.

The free length $x_a$, which depends on the normalized free volume $\beta$, follows the linear mapping $x_a = x^u_a \cdot \beta$. Here, $x^u_a$, determined experimentally to be 40.8 mm, denotes the minimal volume that the filler material occupies in the jammed state. $\beta$ serves as a pivotal parameter in the model, representing the ratio of the current free volume to the maximum free volume within the membrane. It operates as a dynamic entity, delineating the transitions between the gripper’s fluidic and rigid states, thereby facilitating the control and adaptation of the UG to various object geometries. In the s-domain, $\beta$ is described by the equation

2 $$\begin{aligned} \beta (s) = \frac{R(s)}{1+s T}, \end{aligned}$$

with *R*(*s*) serving as a unit step, *s* representing the Laplace variable, and *T* denoting the system’s time constant, approximated as 4.3 s^34^. Variable $\beta$ discerns three discrete states, outlined as

3 $$\begin{aligned} k_{gr}(\beta ) = {\left\{ \begin{array}{ll} \text {closed/jammed} &{} \text {if } \beta \le 1\% , \\ \text {opened/fluidized} &{} \text {if } \beta \ge 99\% , \\ \text {in transition} &{} \text {otherwise}, \end{array}\right. } \end{aligned}$$

highlighting the system’s increased stiffness as $\beta$ approaches zero. This contact model is pivotal in developing a digital copy of the UG for robotic simulators such as *Gazebo*.

### UAV modeling

The optimal trajectory of the AM is determined by balancing multiple objectives:

1. Energy efficiency: Minimize control effort to ensure the path is energetically cost-effective.
2. Visual servoing: Maintain the targeted object within the camera’s field of view for accurate localization.
3. Grasping approach: Initially, maintain a distance $z_{\text {grab}}$ from the floor to prevent collision with the payload and other objects during the approach. Subsequently, when close, descend following a near-vertical path to engage the gripper with the payload.

For the problem visualized in Fig. 1, the following state vector is defined

4 $$\begin{aligned} \pmb {\MakeLowercase {x}} = \left( {{}^{\texttt{W}}\pmb {\MakeLowercase {p}}^{\mathtt {}}_{\mathtt {}}}, {{}^{\texttt{B}}\pmb {\MakeLowercase {v}}^{\mathtt {}}_{\mathtt {}}} \right) \in \mathbb {R}^{8}, \end{aligned}$$

with ${{}^{\texttt{W}}\pmb {\MakeLowercase {p}}^{\mathtt {}}_{\mathtt {}}} = \left( x, y, z, \psi \right)$, and ${{}^{\texttt{B}}\pmb {\MakeLowercase {v}}^{\mathtt {}}_{\mathtt {}}} = \left( v_x, v_y, v_z, v_\psi \right)$, where ${{}^{\texttt{W}}\pmb {\MakeLowercase {p}}^{\mathtt {}}_{\mathtt {}}}$ is the position, $\psi$ the yaw angle of the UAV, and ${{}^{\texttt{B}}\pmb {\MakeLowercase {v}}^{\mathtt {}}_{\mathtt {}}}$ the velocity of the robot. The superscript indicates the frame of reference: $\texttt{W}$ for world and $\texttt{B}$ for body. Lastly, $\pmb {\MakeLowercase {z}}$ is defined as the vector of parameters which includes

5 $$\begin{aligned} \pmb {\MakeLowercase {z}} = \left( {{}^{\texttt{W}}\pmb {\MakeLowercase {p}}^{\mathtt {}}_{\texttt{ref}}}, v_0, v_N, {{}^{\texttt{W}}\pmb {\MakeLowercase {p}}^{\mathtt {}}_{\texttt{vis}}}, c_{lock}, z_{safe}, {{}^{\mathtt {}}\pmb {\MakeLowercase {o}}^{\mathtt {}}_{\texttt{1}}}, \ldots , {{}^{\mathtt {}}\pmb {\MakeLowercase {o}}^{\mathtt {}}_{\mathtt {N_o}}} \right) \in \mathbb {R}^{11+4 N_o}, \end{aligned}$$

with ${{}^{\texttt{W}}\pmb {\MakeLowercase {p}}^{\mathtt {}}_{\texttt{ref}}} = \left( x_{ref}, y_{ref}, z_{ref}, \psi _{ref}\right) ,$ ${{}^{\texttt{W}}\pmb {\MakeLowercase {p}}^{\mathtt {}}_{\texttt{vis}}} = \left( u_{ref}, v_{ref}, w_{ref}\right) ,$ and ${{}^{\mathtt {}}\pmb {\MakeLowercase {o}}^{\mathtt {}}_{\texttt{i}}} = \left( o_{x,i}, o_{y,i}, o_{sx, i}, o_{sy, i}\right) ,$ where ${{}^{\texttt{W}}\pmb {\MakeLowercase {p}}^{\mathtt {}}_{\texttt{ref}}}$ is the desired position of the UAV, ${{}^{\texttt{W}}\pmb {\MakeLowercase {p}}^{\mathtt {}}_{\texttt{vis}}}$ is the visual target location (point of interest), $v_0$ and $v_N$ are the desired velocity at the start, resp., at the end of the horizon, $c_{lock}$ is the binary state indicating whether the visual lock has been acquired or not (i.e., whether a visual target is present), $z_{safe}$ designates the safety altitude and $o_{(\cdot ),i}$ represents the *xy*-centerpoint and minor and major axis of the *i*’th (out of $N_o$) ellipsoidal obstacles. The separation of the visual target and the desired position in combination with $c_{lock}$ gives a great deal of flexibility. By having ${{}^{\texttt{W}}\pmb {\MakeLowercase {p}}^{\mathtt {}}_{\texttt{ref}}} \ne {{}^{\texttt{W}}\pmb {\MakeLowercase {p}}^{\mathtt {}}_{\texttt{vis}}}$, the AM can be tasked to explore the vicinity of the object of interest while keeping it in the field of view of the sensor. At the same time, the sensor lock can be disabled by setting $c_{lock}=0$ to prevent the controller from tracking the target in certain cases (e.g., when moving to the drop-off area). For the actual grasping, ${{}^{\texttt{W}}\pmb {\MakeLowercase {p}}^{\mathtt {}}_{\texttt{ref}}} \approx {{}^{\texttt{W}}\pmb {\MakeLowercase {p}}^{\mathtt {}}_{\texttt{vis}}}$ as the visual target position generally corresponds to the payload position.

Regarding the state dynamics, we propose a basic kinematic UAV hover model, that represents a great simplification of the actual UAV dynamics. High-performance applications generally require more faithful models, however, our main concern is to generate a feasible trajectory that the relatively slow-flying UAV can track. This simple model has the advantage of being easily identifiable and relatively lightweight from a computational point of view. The state dynamics are thus defined as follows:

6 $$\begin{aligned} f\left( \pmb {\MakeLowercase {v}}, \pmb {\MakeLowercase {u}}\right) = {\left\{ \begin{array}{ll} {{}^{\texttt{W}}\dot{\pmb {\MakeLowercase {p}}}^{\mathtt {}}_{\mathtt {}}} &{}= \begin{pmatrix} {}^{\texttt{W}}\pmb {\MakeUppercase {R}}_{\texttt{B}}\left( \psi \right) {{}^{\texttt{B}}\pmb {\MakeLowercase {v}}^{\mathtt {}}_{\mathtt {[xyz]}}} \\ {{}^{\texttt{B}}\pmb {\MakeLowercase {v}}^{\mathtt {}}_{\mathtt {[\psi ]}}} \end{pmatrix}, \\ {{}^{\texttt{B}}\dot{\pmb {\MakeLowercase {v}}}^{\mathtt {}}_{\mathtt {}}} &{}= \frac{1}{\pmb {\MakeLowercase {\tau }}} \left( \pmb {\MakeLowercase {u}} \odot \pmb {\MakeLowercase {k}} - {{}^{\texttt{B}}\pmb {\MakeLowercase {v}}^{\mathtt {}}_{\mathtt {}}}\right) , \end{array}\right. } \end{aligned}$$

where ${}^{\texttt{W}}\pmb {\MakeUppercase {R}}_{\texttt{B}} \in SO(3)$ is the rotation from frame $\texttt{B}$ to $\texttt{W}$, $\odot$ designates the component-wise vector multiplication and the subscript in brackets indicates the components of the respective vector, e.g., ${{}^{\texttt{B}}\pmb {\MakeLowercase {v}}^{\mathtt {}}_{\mathtt {[xyz]}}} \in \mathbb {R}^3$ indicates the *x*, *y*, *z* components of ${{}^{\texttt{B}}\pmb {\MakeLowercase {v}}^{\mathtt {}}_{\mathtt {}}} \in \mathbb {R}^4$.

The gain $k_i$ and time constants $\tau _i$ of the system were determined from the simulated AM with classical step response tangent method:

7a $$\begin{aligned} \tau _i&= \frac{3}{2} \left( t_{y=63\%}-t_{y=28\%}\right) , \end{aligned}$$

7b $$\begin{aligned} k_i&= \frac{y(\infty )}{u(\infty )}. \end{aligned}$$

The results of the system identification are given in Table 1.

**Table 1:** Identified time constants and gains of the simulated first-order AM system.

direction	$k_i$	$\tau _i$ (s)
*x*	1.0	0.51
*y*	1.0	0.51
*z*	1.0	0.40
$\psi$	1.0	0.54

## Trajectory optimization using model predictive control

**Figure 3:**
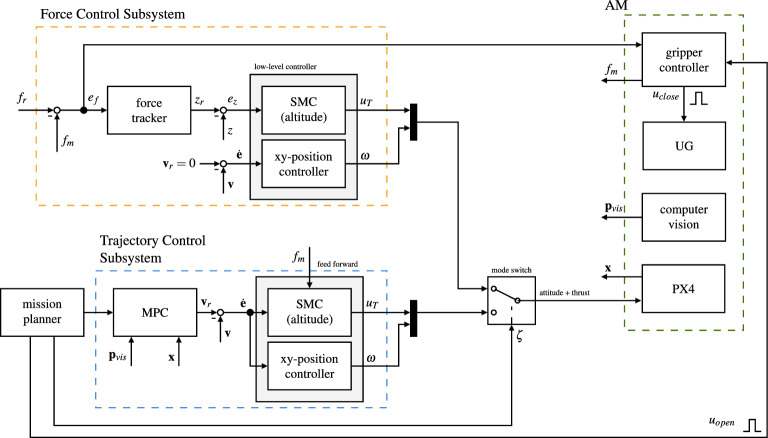
The control architecture consists of a force control subsystem and the MPC trajectory generator orchestrated by the mission planner (a state machine). Depending on the state of the mission, $\zeta$ switches between the *force tracking* and the *trajectory tracking* control modes. Both control modes share a common low-level controller made from the sliding-mode altitude controller and the *xy*-position controller (cascading PID controller). The force controller commands a cumulative force $u_T$ to minimize the error $e_f$ via an intermediate altitude error $e_z$. The trajectory controller feeds velocity commands to the low-level controller. Furthermore, it takes the measured weight of the payload as a feed-forward signal to quickly adapt to the changed dynamics. The gripper controller closes the gripper by emitting $u_{close}$ once a steady state is reached. The mission planner keeps track of all states and executes the mission.

The MPC-based trajectory generation subsystem calculates the optimal velocity commands based on the cost function *J* developed hereafter. This subsystem is embedded in the AM controller as shown in Fig. 3.

### Problem formulation

The discrete-time optimization problem formulation is given by^49^:

8a $$\begin{aligned}{} \underset{\pmb {\MakeLowercase {x}}, \pmb {\MakeLowercase {u}}}{\text {Minimize}}\, \sum _n J(\pmb {\MakeLowercase {x}}_n, \pmb {\MakeLowercase {u}}_n) + E(\pmb {\MakeLowercase {x}}_N){} & {} \end{aligned}$$

8b $$\begin{aligned} {\text{ subject } \text{ to: }}\\{} & {} \pmb {\MakeLowercase {x}}(0)= \pmb {\MakeLowercase {x}}_0&\end{aligned}$$

8c $$\begin{aligned}{} & {} \pmb {\MakeLowercase {x}}_{n+1} = f\left( \pmb {\MakeLowercase {x}}_n, \pmb {\MakeLowercase {u}}_n, t\right) \end{aligned}$$

8d $$\begin{aligned}{} & {} \pmb {\MakeLowercase {u}}_n \in U _n \end{aligned}$$

8e $$\begin{aligned}{} & {} H_n\left( \pmb {\MakeLowercase {x}}_n, \pmb {\MakeLowercase {u}}_n\right) \le 0 \end{aligned}$$

8f $$\begin{aligned}{} & {} H_N\left( \pmb {\MakeLowercase {x}}_N\right) \le 0 \end{aligned}$$

8g $$\begin{aligned}{} & {} n \in \left\{ 1, \dots , N \right\} \end{aligned}$$

where *J* and *E* are the stage and terminal costs, respectively. $H_n$ and $H_N$ represent general inequality constraints, *f* are the system dynamics (6), *n* is the stage number with a total of *N* stages in the control horizon, and $\pmb {\MakeLowercase {u}}$ are the control inputs.

Within this work, the proximal averaged Newton-type method for optimal control (PANOC) algorithm^49^ is used to solve the non-convex optimal control problem (OCP) that is part of the non-linear MPC formulation. The PANOC algorithm solves the fixed-point equations with a fast converging line-search, single direct shooting method. The algorithm, being a first-order method, is particularly well suited for embedded onboard applications with limited memory and compute power and generally outperforms sequential quadratic programming (SQP) methods.

#### Remark 1

The solution to problem (8) is only optimal within the fixed-length moving horizon. Globally seen, the output, i.e., the trajectory, can be suboptimal, even with a risk of getting trapped in a local minimum.

### Cost function: perception

**Figure 4:**
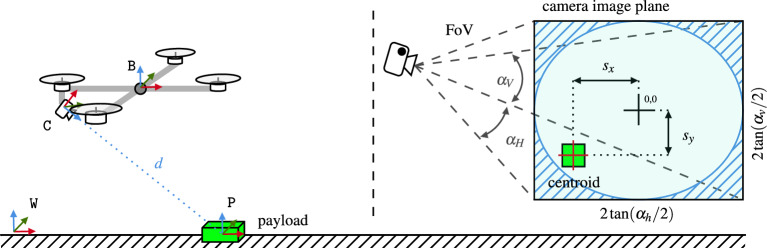
The UAV’s camera detects the payload in green and assigns it a position within its frame of reference. The centroid of the payload is projected back into the camera’s image plane, where it is assigned the coordinates $s_x$ and $s_y$.

For a safe and reliable approach, the targeted object should preferably stay in the field of view (FoV) of the AM. Herein, we follow the development in^50^ to impose this behavior on the AM. Imposing this as a hard constraint would in many cases lead to an infeasible problem. By softening those constraints, the MPC gets the flexibility to find a solution that is a more favorable compromise between the objectives, e.g., saving energy at the expense of slightly violating the perception constraint.

The violation of a constraint is a binary state; either it is violated ’1’, or not ’0’. If it is, there is a non-zero cost associated with it, which incentivizes the optimizer to find a solution that satisfies the constraint. That binary behavior is akin to the Heaviside step function

9 $$\begin{aligned} H(x) = {\left\{ \begin{array}{ll} 1, &{} x > 0,\\ 0, &{} \text {otherwise}, \end{array}\right. } \end{aligned}$$

However, its discontinuous nature makes it unsuitable for numerical optimization. Better candidates are found in the family of logistic functions. Herein we use the sigmoid function instead of the Heaviside step function as it is continuously differentiable over its entire domain. The sigmoid function is defined as

10 $$\begin{aligned} \sigma \left( x, x_0, k_s, k_g\right) = \frac{k_g}{1+\exp \left( -k_s \left( x-x_0\right) \right) }, \end{aligned}$$

where $k_s$ defines the steepness of the transition between 0 and $k_g$. The parameter $x_0$ defines the center point of the transition, i.e., $\sigma \left( x, x_0, k_s, k_g\right) =k_g/2$ for $x=x_0$. Based on the sigmoid function, the unit pulse function is defined as

11 $$\begin{aligned} \text {pulse}\left( x, k_s, k_g\right) = 4 k_g \sigma \left( x, x_0=0, k_s, k_g=1\right) \left( 1- \sigma \left( x, x_0=0, k_s, k_g=1\right) \right) . \end{aligned}$$

To satisfy the perception constraints, the MPC has to ensure that the centroid of the target object stays within the image plane of the camera as depicted in Fig. 4. The formulation herein does not force the MPC to keep the target in the center of the image plane and thus avoids unnecessary movements, keeping the AM more stable.

The object detection pipeline provides the position of the target object in world coordinates denoted as ${{}^{\texttt{W}}\pmb {\MakeLowercase {p}}^{\mathtt {}}_{\texttt{vis}}}$, resp., in local camera frame $\texttt{C}$ as ${{}^{\texttt{C}}\pmb {\MakeLowercase {p}}^{\mathtt {}}_{\texttt{vis}}} = \left( {}^{\texttt{W}}\pmb {\MakeUppercase {T}}_{\texttt{B}} {}^{\texttt{B}}\pmb {\MakeUppercase {T}}_{\texttt{C}}\right) ^{-1} {{}^{\texttt{W}}\pmb {\MakeLowercase {p}}^{\mathtt {}}_{\texttt{vis}}}$, with ${}^{\mathtt {}}\pmb {\MakeUppercase {T}}_{\mathtt {}} \in SE(3)$ being the transformation matrix between the frames in super-, resp., subscript. Its coordinates $\pmb {\MakeLowercase {s}}$ in the virtual image plane are then obtained with the help of the following relationship:

12 $$\begin{aligned} \pmb {\MakeLowercase {s}}\left( {{}^{\texttt{C}}\pmb {\MakeLowercase {p}}^{\mathtt {}}_{\texttt{vis}}}\right) = \begin{pmatrix} {{}^{\texttt{C}}\pmb {\MakeLowercase {p}}^{\mathtt {}}_{\texttt{vis}}}_{[x]} / {{}^{\texttt{C}}\pmb {\MakeLowercase {p}}^{\mathtt {}}_{\texttt{vis}}}_{[z]} \\ {{}^{\texttt{C}}\pmb {\MakeLowercase {p}}^{\mathtt {}}_{\texttt{vis}}}_{[y]} / {{}^{\texttt{C}}\pmb {\MakeLowercase {p}}^{\mathtt {}}_{\texttt{vis}}}_{[z]} \end{pmatrix}. \end{aligned}$$

Equation (12) has a singularity at ${{}^{\texttt{C}}\pmb {\MakeLowercase {p}}^{\mathtt {}}_{\texttt{vis}}}_{[z]} = 0$. In practice, this is not an issue since the distance from the camera to the target can never be zero (the RealSense D435 has a minimal distance of 8 cm). Taking this into account, Eq. (12) is conditioned to eliminate the singularity at ${{}^{\texttt{C}}\pmb {\MakeLowercase {p}}^{\mathtt {}}_{\texttt{vis}}}_{[z]}=0$ as follows:

13a $$\begin{aligned} \pmb {\MakeLowercase {s}}\left( {{}^{\texttt{C}}\pmb {\MakeLowercase {p}}^{\mathtt {}}_{\texttt{vis}}}\right)&= \begin{pmatrix} {{}^{\texttt{C}}\pmb {\MakeLowercase {p}}^{\mathtt {}}_{\texttt{vis}}}_{[x]} / z^* \\ {{}^{\texttt{C}}\pmb {\MakeLowercase {p}}^{\mathtt {}}_{\texttt{vis}}}_{[y]} / z^* \end{pmatrix}, \end{aligned}$$

13b $$\begin{aligned} z^*&= {{}^{\texttt{C}}\pmb {\MakeLowercase {p}}^{\mathtt {}}_{\texttt{vis}}}_{[z]} + s_p, \end{aligned}$$

where $s_p = \text {pulse}\left( x={{}^{\texttt{C}}\pmb {\MakeLowercase {p}}^{\mathtt {}}_{\texttt{vis}}}_{[z]}, x_0=0, k_s=80, k_g\right)$ and therefore $\lim _{{{}^{\texttt{C}}\pmb {\MakeLowercase {p}}^{\mathtt {}}_{\texttt{vis}}}_{[z]} \rightarrow \pmb {\MakeLowercase {0}}} z^* = k_g$, with $k_g$ being a very small positive number.

The inequality constraints that keep $\pmb {\MakeLowercase {s}}$ in the FoV of the camera (defined by $\alpha _h$ and $\alpha _v$) are thus $|{\pmb {\MakeLowercase {s}}_{[x]}}| < \tan {\frac{\alpha _h}{2}}$ and $|{\pmb {\MakeLowercase {s}}_{[y]}}| < \tan {\frac{\alpha _v}{2}}$, or more conveniently expressed as an inequality that restricts $\pmb {\MakeLowercase {s}}$ to lay within an ellipsoid centered around $\pmb {\MakeLowercase {0}}$ defined by its minor and major axis $h=\tan \alpha _h$ and $w=\tan \alpha _v$ such that

14 $$\begin{aligned} \frac{\pmb {\MakeLowercase {s}}_{[x]}^2}{(w/2)^2} + \frac{\pmb {\MakeLowercase {s}}_{[y]}^2}{(h/2)^2} < 1. \end{aligned}$$

By making use of the sigmoid logistic function, (14) becomes

15 $$\begin{aligned} c_{p,1}\left( {{}^{\texttt{C}}\pmb {\MakeLowercase {p}}^{\mathtt {}}_{\texttt{vis}}}\right) = \sigma \left( \frac{\pmb {\MakeLowercase {s}}_{[x]}^2}{(w/2)^2} + \frac{\pmb {\MakeLowercase {s}}_{[y]}^2}{(h/2)^2}, 1, k_{s1}, k_{g1} \right) . \end{aligned}$$

Eq. (15) has, however, two problems; *first*, it represents a double elliptical cone, i.e., the constraint is satisfied even if the target is behind the camera, *second*, ${\nabla c_{p,1}}\approx 0$ in regions where the constraint is violated, which makes recovering from constraint violations unlikely. The solution to the first problem is to add a constraint that guarantees ${{}^{\texttt{C}}\pmb {\MakeLowercase {p}}^{\mathtt {}}_{\texttt{vis}}}_{[z]} > 0$ (target in front of the camera), resp. $c_{p,z} = \sigma \left( -{{}^{\texttt{C}}\pmb {\MakeLowercase {p}}^{\mathtt {}}_{\texttt{vis}}}_{[z]}, 0, k_{s2}, k_{g2}\right)$, which extends (15) to become

16 $$\begin{aligned} c_{p,2}\left( {{}^{\texttt{C}}\pmb {\MakeLowercase {p}}^{\mathtt {}}_{\texttt{vis}}}\right) = c_{p,z} + c_{p,1} \left( 1-c_{p,z}\right) . \end{aligned}$$

Lastly, a quadratic term and a bias term are added to the formulation such that the gradient of non-compliant regions fulfills ${\nabla c_{p}} > 0$:

17 $$\begin{aligned} c_{p}\left( {{}^{\texttt{C}}\pmb {\MakeLowercase {p}}^{\mathtt {}}_{\texttt{vis}}}\right) = c_{p,2} \left( 1+k_g \left\| { \begin{pmatrix} {{}^{\texttt{C}}\pmb {\MakeLowercase {p}}^{\mathtt {}}_{\texttt{vis}}}_{[x]} \\ {{}^{\texttt{C}}\pmb {\MakeLowercase {p}}^{\mathtt {}}_{\texttt{vis}}}_{[y]} \\ c_{p,z} {{}^{\texttt{C}}\pmb {\MakeLowercase {p}}^{\mathtt {}}_{\texttt{vis}}}_{[z]} \end{pmatrix} }\right\| ^2 + c_{p,z} \cdot \text {pulse}\left( \left( {{}^{\texttt{C}}\pmb {\MakeLowercase {p}}^{\mathtt {}}_{\texttt{vis}}}_{[x]}\right) ^2, 0, k_{s3}, k_{g3}\right) \right) . \end{aligned}$$

The quadratic term includes the condition $c_{p,z}$ such that the distance of the target from the camera in the positive *z*-direction is not penalized. The bias term in form of the pulse function gives the optimizer an extra incentive to turn the UAV towards the target. The constraint function (17) is included as perception cost function

18 $$\begin{aligned} J_P\left( \pmb {\MakeLowercase {x}}\right) = k_P \cdot c_{lock} \cdot c_p, \end{aligned}$$

in (8), where $k_P$ is a positive weight and $c_{lock} \in \left\{ 0,1\right\}$ accommodates for the case where no target is detected, and the perception cost should consequently be ignored. This also accounts for cases where the UAV loses sight of the visual target, e.g., due to an obstacle blocking the line of sight with the target.

### Cost function: tracking

During approach and departure, the AM is commanded to reach a given target position ${{}^{\texttt{W}}\pmb {\MakeLowercase {p}}^{\mathtt {}}_{\texttt{ref}}}$ and yaw angle $\psi _{ref}$ with its current position and orientation given by ${{}^{\texttt{W}}\pmb {\MakeLowercase {p}}^{\mathtt {}}_{\mathtt {}}}$ and $\psi$. Herein, a cost function is created that encourages the UAV to approach the commanded location for translation and rotation, starting with the latter.

The yaw angle error $\psi _{err} = \psi _{ref} - \psi$ is not a useful quantity to feed into the OCP due to the discontinuity at ${0}^\circ$ resp. ${360}^\circ$. Instead, the orientation is encoded in the two-dimensional direction vector

19 $$\begin{aligned} \pmb {\MakeLowercase {q}}\left( \psi \right) = \begin{pmatrix} \cos \psi \\ \sin \psi \end{pmatrix}, \end{aligned}$$

with the orientation error being

20 $$\begin{aligned} {{}^{\mathtt {}}\pmb {\MakeLowercase {q}}^{\mathtt {}}_{\texttt{err}}}\left( \pmb {\MakeLowercase {x}}\right) = \frac{1}{2} \left( {{}^{\mathtt {}}\pmb {\MakeLowercase {q}}^{\mathtt {}}_{\texttt{ref}}}\left( \psi _{ref}\right) - \pmb {\MakeLowercase {q}}\left( \psi \right) \right) . \end{aligned}$$

Likewise, the position error is defined as

21 $$\begin{aligned} {{}^{\mathtt {}}\pmb {\MakeLowercase {p}}^{\mathtt {}}_{\texttt{err}}} = {{}^{\texttt{W}}\pmb {\MakeLowercase {p}}^{\mathtt {}}_{\texttt{ref}}} - {{}^{\texttt{W}}\pmb {\MakeLowercase {p}}^{\mathtt {}}_{\mathtt {}}}. \end{aligned}$$

Lastly, to have some control over the velocity at the start $v_0$ and the end $v_N$ of the horizon, the following quantity is defined

22a $$\begin{aligned} v_{ref}&= \left( 1-\alpha \right) v_0 + \alpha v_N, \end{aligned}$$

22b $$\begin{aligned} v_{err}&= \left\| {{{}^{\texttt{B}}\pmb {\MakeLowercase {v}}^{\mathtt {}}_{\mathtt {}}}}\right\| - v_{ref}, \end{aligned}$$

22c $$\begin{aligned} \alpha&= n/N, \end{aligned}$$

where *N* is the number of states, and *n* is the current stage. The velocity at each stage of the moving horizon is changed linearly between $v_0$ at the beginning to $v_N$ at the end to incentivize a gradual (linear) change of velocity and thus avoid an aggressive braking maneuver when approaching the commanded location.

The cost function $J_T$ associated with the position tracking is composed by (19), (22) and (21). To account for the varying objectives of that cost function, $c_{lock}$ is defined to switch between either the tracking of the reference angle or the targeted object via the perception constraint. The cost equation to include in (8) is therefore

23 $$\begin{aligned} J_T\left( \pmb {\MakeLowercase {x}}\right) = k_{T,1} \left\| {{{}^{\mathtt {}}\pmb {\MakeLowercase {p}}^{\mathtt {}}_{\texttt{err}}}}\right\| ^2 + k_{T,2} v_{err}^2 + k_{T,3} \dot{\psi }^2 + \left( 1-c_{lock}\right) k_{T,4} \left\| {{{}^{\mathtt {}}\pmb {\MakeLowercase {q}}^{\mathtt {}}_{\texttt{err}}}}\right\| ^2, \end{aligned}$$

where $k_{T,i}$ are positive weights.

### Cost function: grasping

The grasping cost function takes the particularities of the UG into account; it guarantees that the gripper stays at a safe altitude from the ground as long as it is not located above the target, and it only allows the AM to descend while being in close vicinity. At the same time, if the UAV for some reason, e.g., a wind gust, gets dislocated from the target’s location, it is forced to climb again. For that reason, two constraint functions are defined. The altitude penalty constraint is defined as

24 $$\begin{aligned} c_4 = 1 - \sigma \left( z, z_{min}, k_{s4}, k_{g4}\right) , \end{aligned}$$

where $z_{min}$ marks the safe minimal altitude the AM has to keep. However, as this would prevent the AM from descending to the payload, an additional term is needed based on the *xy*-position relative to the target, i.e.,

25 $$\begin{aligned} c_5 = 1-\text {pulse}\left( \left\| {{{}^{\texttt{W}}\pmb {\MakeLowercase {p}}^{\mathtt {}}_{\texttt{ref}}}_{[xy]} - {{}^{\texttt{W}}\pmb {\MakeLowercase {p}}^{\mathtt {}}_{\mathtt {}}}_{[xy]}}\right\| ^2, k_{s5}, k_{g5} \right) , \end{aligned}$$

where $k_s$ is chosen as a function of the radius *r* of the cone through which the UAV is funneled to the target. Herein $k_s\left( r={0.1} \text{m}\right) =176.27$ was determined from solving the following equations:

26a $$\begin{aligned} x_{0.5}(k_s)&:=1-\text {pulse}\left( x, k_s, k_g=1\right) = 0.5 \end{aligned}$$

26b $$\begin{aligned} k_s(r)&:=x_{0.5}(k_s) = r^2. \end{aligned}$$

The pulse function has the particularity of $\text {pulse}\left( x, k_s, k_g\right) =0$ at $x=0$, resulting in no residual cost at ${{}^{\texttt{W}}\pmb {\MakeLowercase {p}}^{\mathtt {}}_{\texttt{ref}}}_{xy} = {{}^{\texttt{W}}\pmb {\MakeLowercase {p}}^{\mathtt {}}_{\mathtt {}}}_{xy}$. The total cost associated with the grasping constraints is thus defined as

27 $$\begin{aligned} J_G\left( \pmb {\MakeLowercase {x}}\right) = k_{G,1} c_4 c_5 \left( 1 + k_{G,2}\left( z_{min}-z\right) ^2\right) + k_{G,3} (1-c_5) \left\| { {{}^{\texttt{B}}\pmb {\MakeLowercase {v}}^{\mathtt {}}_{\mathtt {}}} }\right\| ^2, \end{aligned}$$

where $k_{G,i}$ are tuneable weights and $k_{G,3} (1-c_5) \left\| { {{}^{\texttt{B}}\pmb {\MakeLowercase {v}}^{\mathtt {}}_{\mathtt {}}} }\right\| ^2$ penalizes for any velocity close to the target.

### Cost function: repulsion

In many applications, it is useful to define areas that should be avoided during flight, e.g., pedestrians, lamp posts, walls or cars. Herein, it is assumed that those areas can be approximated with one or multiple ellipsoids. It is assumed that the targets are static, localized, and cannot be overflown (for safety resp. physical reasons), thus resulting in a 2D *xy*-problem.

On that account, an axis-aligned ellipsis is defined, located at the world position $\left( o_x, o_y\right)$ with its minor and major axis defined by $\left( o_{sx}, o_{sy}\right)$. A point $\left( p_x, p_y\right)$ is inside the ellipsis if

28 $$\begin{aligned} \frac{\left( p_x - o_x\right) ^2}{\left( o_{sx}/2\right) ^2} + \frac{\left( p_y - o_y\right) ^2}{\left( o_{sy}/2\right) ^2} < 1. \end{aligned}$$

Reformulating that expression using the sigmoid logistic function yields

29 $$\begin{aligned} c_R = 1 - \sigma \left( \frac{\Delta p_x^2}{\left( o_{sx}/2\right) ^2} + \frac{\Delta p_y^2}{\left( o_{sy}/2\right) ^2}, 1, k_{s6}, k_{g6}\right) . \end{aligned}$$

The repulsion cost function for a single area of repulsion *i* is thus

30 $$\begin{aligned} J_{R,i} = c_{R,i} \cdot \sum \left( \begin{pmatrix} o_{sx,i}^2 \\ o_{sy,i}^2 \end{pmatrix} - \begin{pmatrix} \Delta p_{x,i}^2 \\ \Delta p_{y,i}^2 \end{pmatrix} \right) , \end{aligned}$$

where a quadratic term was added to improve the gradient in the non-compliant region.

The total repulsion cost of all areas is, therefore

31 $$\begin{aligned} J_R\left( \pmb {\MakeLowercase {x}}\right) = k_R \sum _i^{N_o} J_{R,i}. \end{aligned}$$

Herein (31) is included as a soft constraint, rather than as a hard constraint. This has the advantage of being computationally cheaper but may lead to collisions if the repulsion cost term is dominated by other cost functions.

An environment may contain hundreds of obstacles and including each obstacle in (5) makes solving the OCP computationally very expensive. However, there are generally only a handful of obstacles near the UAV that must be considered. Therefore by simply feeding the momentarily closest obstacles into the OCP’s parameter vector, a large environment with many obstacles can be considered with $N_o$ still being reasonably small (here, $N_o=2$). Furthermore, the AM’s body size can be accounted for by simply expanding the ellipsis of the obstacle by the radius $r_B$ of the body, i.e., $\left( \hat{o}_{sx}, \hat{o}_{sy}\right) = \left( o_{sx} + r_B, o_{sy} + r_B\right)$. Finally, an obstacle with a non-ellipsoidal shape can be approximated by placing several ellipses within its contour.

## Force control

For optimal operation, the UG requires the activation force to be controlled, i.e., kept constant, over the grasping interval. More precisely, the force should not exceed a certain level as it may cause damage to the gripper (or to the payload), and it must also not drop below a certain threshold as this servery degrades the grasping performance, as discussed in our previous work^34^.

The fundamental problem is thus to control the contact force, as it was shown in^51^ and^52^ for bridge inspection and in^53^ in the context of aerial writing. The noticeable difference here is the strong nonlinearity of the elastic element and the shrinkage of the membrane, which consequently changes the stiffness of the system during operation and may also cause the complete loss of contact. The cascading force control approach followed herein is inspired by^54^, and^55^. Since the contact force measured by the load-cell and the position of the UAV are linked, the contact force tracking problem can be seen as a position tracking problem where the reference position is a function of the force tracking error. Motivated by this statement, the cascading control architecture as shown in Fig. 3 is developed.

### Problem formulation

The physical properties and grasping performance of the UG were rigorously analyzed in^34^ with the main conclusion being that the system is highly non-linear and state-dependent. In particular, the stiffness of the elastic element changes from very soft to rigid-like.

**Figure 5:**
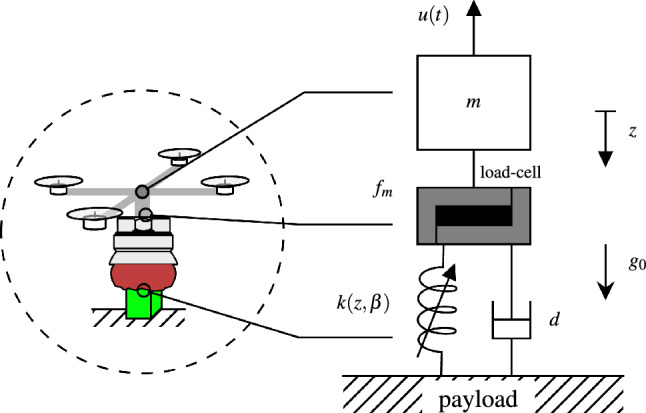
The UG in contact with the payload is modeled as a mass-spring-damper system, where the spring force is non-linear with respect to the penetration depth *z* and further depends on the UG state $\beta$. The contact force is measured by a load-cell between the UG’s membrane and the rest of the AM (represented by *m*). The damping *d* represents the friction of the grains in the filler as well as other effects such as air resistance.

The homologous model of the AM is as shown in Fig. 5 consisting of a mass-spring-damper system, where the stiffness *k* is non-linear, compression-only and dependant on the gripper’s state $\beta$ and depth of entrance *z*. The unknown damping *d* contains various effects such as the friction between the filler particles and air resistance. The mass *m* is the total mass of the AM with $u_T$ being the cumulative thrust applied by the aircraft’s propulsion system.

The dynamics of the system are modeled according to the following set of equations

32a $$\begin{aligned} \ddot{z}&= \frac{1}{m} \left[ -f_{kd}\left( z, \dot{z}\right) \vert _{z>0} + m g_0 - u_T \right] , \end{aligned}$$

32b $$\begin{aligned} z\left( 0\right)&= 0, \end{aligned}$$

32c $$\begin{aligned} \dot{z}\left( 0\right)&= v_0, \hspace{0.1cm} v_0 > 0, \end{aligned}$$

32d $$\begin{aligned} f_{kd}\left( z, \dot{z}\right)&= f_k\left( z\right) + f_d\left( \dot{z}\right) , \end{aligned}$$

where $u_T$ is the input (applied thrust) to the system, the states $\dot{z}$ and *z* are measurable, $f_d\left( z, \dot{z}\right)$ and $f_k\left( z\right)$ are smooth, positive definite functions. It is assumed that the system has an initial downward velocity $\dot{z}=v_0$. The initial position is defined as $z(0)=0$, which marks the position at which the gripper touches the ground. Assuming ground contact, the load-cell measures the contact force $f_m$ at 100 Hz as

33 $$\begin{aligned} f_m = f_{kd}\left( z, v_z\right) . \end{aligned}$$

### Force tracking

The activation force is indirectly tracked by controlling the position (altitude) of the drone. The control solution thus consists of a *force tracker* that feeds into a robust sliding mode *position tracker* (Fig. 3) as explained in the following.

#### Sliding mode position tracker

The position tracker has been realized with a classic sliding mode control (SMC) method^56,57^. The system can be seen as uni-axial (altitude only) since the UAV will be hovering (stationary in *xy*-direction, held in position mostly by friction) during the grasp. The non-linear uni-axial system is considered as

34 $$\begin{aligned} \dot{v}_z = f\left( \pmb {\MakeLowercase {x}}\right) + g\left( \pmb {\MakeLowercase {x}}\right) u\left( t\right) + \vartheta \left( t\right) , \end{aligned}$$

where $f\left( \cdot \right)$ and $g\left( \cdot \right)$ are non-linear smooth functions and $\pmb {\MakeLowercase {x}}$ being the state vector defined in (4). The unknown disturbance is assumed to be bounded $|{\vartheta }| < D$. The sliding surface is defined as

35a $$\begin{aligned} s&= \dot{e_z} - c e_z, \end{aligned}$$

35b $$\begin{aligned} \dot{s}&= \ddot{e_z} - c \dot{e_z}, \end{aligned}$$

where $c>0$ is a design parameter and $e_z=z_r-z$ is the tracking error. Assuming a constant rate reaching-law

36 $$\begin{aligned} \dot{s} = -\eta \cdot \text {sign}\left( s\right) \text {, } \eta > 0, \end{aligned}$$

the input can be defined as

37 $$\begin{aligned} u_{T}=\frac{1}{g} \left( -\eta \cdot \text {sign}\left( s\right) - \ddot{z}_r + f + c\dot{e_z} \right) , \end{aligned}$$

which is stabilizes the system under the condition that $\eta > D$^56^. To reduce chatter, the sign function is replaced by the continuous saturation function^56^, as

38 $$\begin{aligned} u_{T}=\frac{1}{g} \left( -\eta \cdot \text {sat}\left( s\right) - \ddot{z}_r + f + c\dot{e_z} \right) . \end{aligned}$$

With the UAV in hover-position, the uni-axial dynamics can be stated as

39 $$\begin{aligned} f_z = {\left\{ \begin{array}{ll} \dot{v}_z &{}= f\left( \pmb {\MakeLowercase {x}}\right) + g u\left( t\right) + \vartheta \left( t\right) , \\ \dot{z} &{}= v_z, \end{array}\right. } \end{aligned}$$

with $f\left( \pmb {\MakeLowercase {x}}\right) = \frac{1}{m} \left( f_m - g_0 \right) ,$ and $g = \frac{1}{m}.$ Considering the sliding surface as defined in (35a) and the reaching law in (36), the control output $u_T$ of the tracker is defined as

40 $$\begin{aligned} u_{T}\left( t\right) = -f_m + m \left( g_0 - c\dot{e_z} - \eta \cdot \text {sat}\left( s\right) \right) . \end{aligned}$$

#### Force tracker

**Figure 6:**
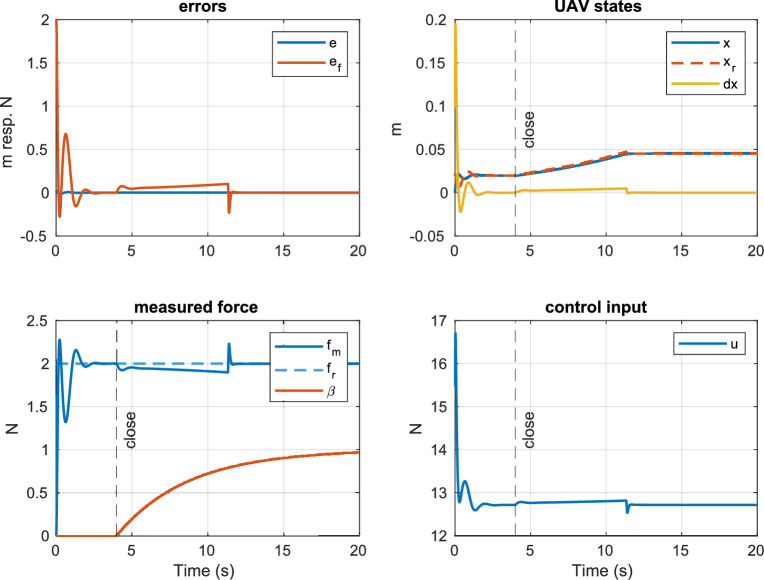
Simulated closed-loop response of the force controller. The gripper is closed once the system reaches steady-state (around the 4 s mark). The chattering on the input $u\left( t\right)$ is effectively mitigated by the saturation function.

The force applied by the UG to the payload, as it is measured by the sensor, is directly dependent on the system states *z* and $\dot{z}$. Therefore, the force can be regulated by commanding and tracking a specific position (altitude) $z_r$. To that goal, the force tracker is defined as

41a $$\begin{aligned} z_r&= k_p e_f + w_{e_f}, \end{aligned}$$

41b $$\begin{aligned} \dot{w}_{e_f}&= k_i e_f, \end{aligned}$$

where $k_p$ and $k_i$ are positive design variables. The gripper is closed once the system has reached steady-state, i.e., $|{e_f}| < c_{e1}$ and $|{\dot{e}_f}| < c_{e2}$ with $c_{e1}$, $c_{e2}$ being small positive values. Closing the gripper triggers its state transition from soft to rigid. The complete control architecture is depicted in Fig. 3. The simulated system response for the closed-loop system with an initial position $z\left( 0\right) ={0}\,\hbox {m}$ and velocity of $\dot{z}\left( 0\right) = {0.1}\,\hbox {ms}^{-1}$ is shown in Fig. 6. The design variables were experimentally chosen as stated in Table 2.

**Table 2:** The empirically tuned constants of the SMC.

Equation	Tuneable constants	Assigned values
SMC (40)	$\left( c, \eta ,\epsilon \right)$	$\left( 3, 4, 0.8 \right)$
Force Tracker (41)	$\left( k_p, k_i \right)$	$\left( 0.03, 0.05 \right)$

##### Remark 2

Herein *z* is also synonymous to the UAV’s altitude relative to the position the gripper first entered in contact with the payload, i.e., the point where $f_m>\delta$ and $\dot{f}_m>0$ is measured ($\delta$ being a small threshold value). By definition, the initial position *z*(0) is thus zero. An initial velocity $\dot{z}(0)>0$ is required for the AM to engage with the payload (commanded by the MPC).

The gripper controller sends the close command around the $t={4}\,\hbox {s}$ mark as the closed system reaches steady-state. This triggers the UG’s state transition from soft to rigid during which the membrane shrinks, and consequently, the UAV has to descend to compensate for the diminishing contact force.

## Numerical validation and virtual experiment

### Numerical validation

The objective of this section is to verify that the developed cost functions produce the desired outcomes and behaviors. To that aim the developed cost functions are included in (8) as the sum of the individual cost functions for perception (18), position tracking (23), grasping (27), repulsion (31), and an additional term $J_U$ that penalizes for energy-inefficient trajectories:

42 $$\begin{aligned} J\left( \pmb {\MakeLowercase {x}}, \pmb {\MakeLowercase {u}}\right) = J_P\left( \pmb {\MakeLowercase {x}}\right) + J_T\left( \pmb {\MakeLowercase {x}}\right) + J_G\left( \pmb {\MakeLowercase {x}}\right) + J_R\left( \pmb {\MakeLowercase {x}}\right) + J_U\left( \pmb {\MakeLowercase {u}}\right) . \end{aligned}$$

The quadratic control effort penalty is defined as

43 $$\begin{aligned} J_U\left( \pmb {\MakeLowercase {u}}\right) = \pmb {\MakeLowercase {u}}^T \pmb {\MakeUppercase {Q}}_u \pmb {\MakeLowercase {u}}. \end{aligned}$$

A potential issue can arise as a consequence of using soft constraints on the objectives (18), (27) and (31) where any non-bounded cost in (42) can dominate all other objectives, which, ultimately, leads to a violation of the soft constraints (in particular the repulsion constraint). Herein, the position tracking cost (23) represents such a non-bounded cost. Therefore, our implementation includes a carrot-chasing law for position tracking that feeds a virtual target location ${{}^{\texttt{W}}\pmb {\MakeLowercase {p}}^{\mathtt {}}_{\texttt{ref,virt}}}$ to the MPC that cannot exceed a certain distance from the UAV’s current position. This effectively means that (23) is bounded, regardless of the actual distance to the target. The virtual target displacement $\pmb {\MakeLowercase {w}}$ is defined as follows

44a $$\begin{aligned} \pmb {\MakeLowercase {w}}&= {{}^{\texttt{W}}\pmb {\MakeLowercase {p}}^{\mathtt {}}_{\texttt{ref,virt}}} - {{}^{\texttt{W}}\pmb {\MakeLowercase {p}}^{\mathtt {}}_{\mathtt {}}}, \end{aligned}$$

44b $$\begin{aligned} \left\| {\pmb {\MakeLowercase {w}}}\right\|&\le w_{max}, \end{aligned}$$

44c $$\begin{aligned} w_{max}&= N \cdot t_s \cdot v_{max}. \end{aligned}$$

where $w_{max}$ represents the maximum distance that can be covered during the moving horizon at speed $v_{max}$, *N* is the number of stages in the horizon, and $t_s$ is the time step. The virtual target point ${{}^{\texttt{W}}\pmb {\MakeLowercase {p}}^{\mathtt {}}_{\texttt{ref,virt}}}$ is fed to the MPC as ${{}^{\texttt{W}}\pmb {\MakeLowercase {p}}^{\mathtt {}}_{\texttt{ref}}} = {{}^{\texttt{W}}\pmb {\MakeLowercase {p}}^{\mathtt {}}_{\texttt{ref,virt}}}$ with the reference velocities $v_0$, and $v_N$ defined as follows

45 $$\begin{aligned} v_0&= \alpha \cdot v_{max}, \end{aligned}$$

46 $$\begin{aligned} v_N&= {\left\{ \begin{array}{ll} 0 &{}\text {if } \left\| {\pmb {\MakeLowercase {w}}}\right\| < w_{max}, \\ v_{max} &{}\text {otherwise}, \end{array}\right. } \end{aligned}$$

47 $$\begin{aligned} \alpha&= \left\| {\pmb {\MakeLowercase {w}}}\right\| / w_{max} \in \left[ 0;1\right] . \end{aligned}$$

The UAV is thus encouraged to travel at maximum speed if the virtual target is located at a distance $\left\| {\pmb {\MakeLowercase {w}}}\right\| \ge w_{max}$, i.e., the target cannot be reached within the horizon. Once the target gets closer ($\left\| {\pmb {\MakeLowercase {w}}}\right\| < w_{max}$), the UAV has to slow down and is incentivized to reach zero velocity at the end of the moving horizon. The initial reference velocity $v_0$ is gradually reduced as the robot gets closer to the target point.

The developed MPC is validated numerically in three different scenarios, testing the perception, tracking, grasping, and obstacle avoidance components. The implementation uses the Optimal Control Problem (OCP) described in (8) within the OpEn^58^ framework that also manages code generation and auto-differentiation using CasADi^59^, and it employs the fast PANOC solver for embedded applications.

As indicated in^60^, the prediction horizon must be chosen carefully such that a change to the input $\pmb {\MakeLowercase {u}}$ has a chance to realize an effect on the system states. Herein, the duration of the moving horizon is therefore based on the slowest subsystem of (6), which has the time constant $\tau = {0.54}\,\hbox {s}$ (see Table 1). Since a first order system reaches steady-state after $5\tau$, and given an adequate sampling interval of $T_s = {0.1}\,\hbox {s}$, the number of stages can be calculated as

48 $$\begin{aligned} N = \frac{5\tau }{T_s} = 26. \end{aligned}$$

The simulation performed inside MATLAB applies the first set of inputs from the MPC to the model defined in (6) and updates the system states using the forward Euler integration scheme. The empirically tuned weights of the MPC used throughout the simulated scenarios are as indicated in Table 3.

**Table 3:** The empirically tuned gains of the MPC.

Cost function	Tuneable constants	Assigned values
Perception (18)	$\left( k_{s1}, k_{g1}, k_{s2}, k_{g2}, k_{s3}, k_{g3} \right)$	$\left( 1, 5, 0, 30, 1, 20 \right)$
Tracking (23)	$\left( k_{T,1}, k_{T,2}, k_{T,3}, k_{T,4} \right)$	$\left( 10, 20, 10, 100 \right)$
Grasping (27)	$\left( k_{G,1}, k_{G,2}, k_{G,3}, k_{s4}, k_{g4}, k_{s5}, k_{g5} \right)$	$\left( 50, 1, 80, 20, 1, 0, 176.2747 \right)$
Obstacle (31)	$\left( k_{R}, k_{s6}, k_{g6} \right)$	$\left( 1, 1, 20\right)$
Control effort (43)	$\pmb {\MakeUppercase {Q}}_u$	$\left( 10, 10, 10, 10\right)$

#### Scenario 1 (perception, inside FoV)

**Figure 7:**
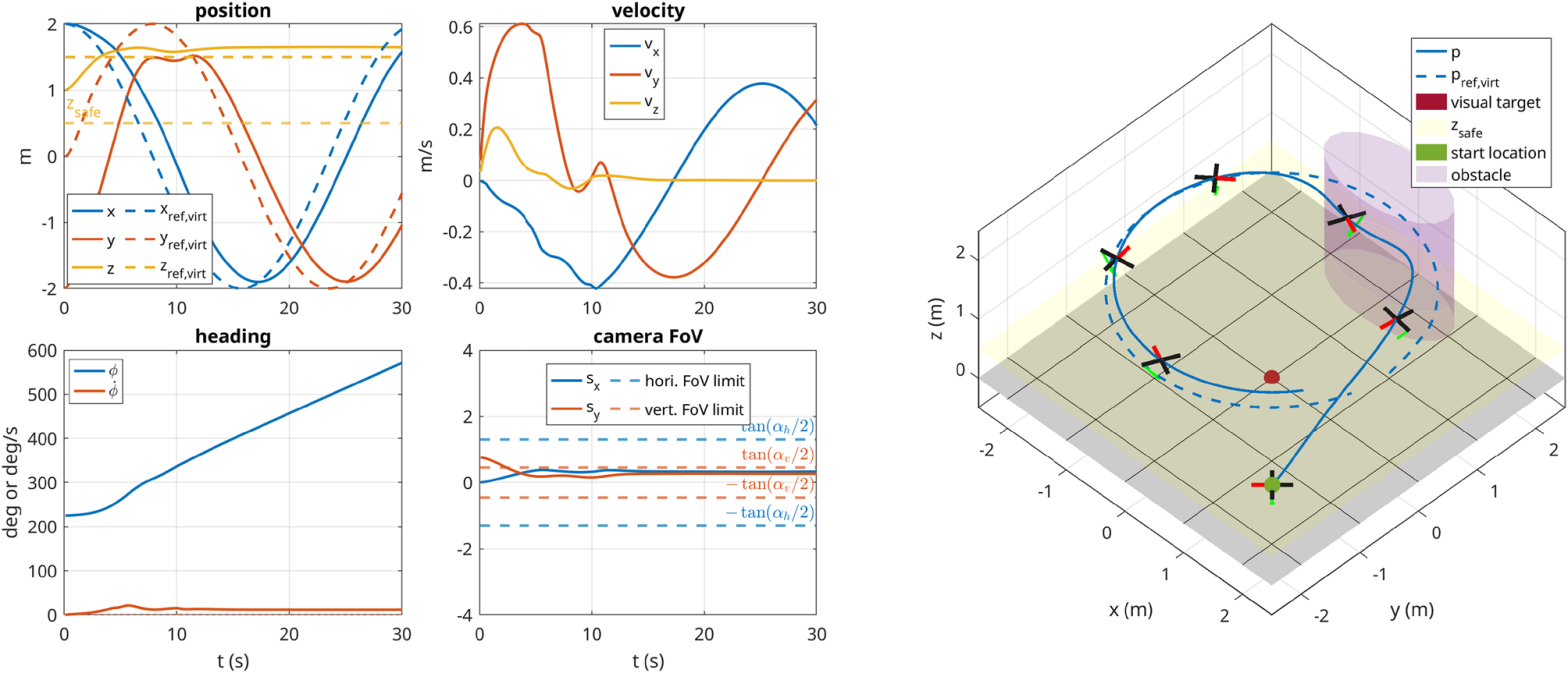
Scenario 1: The UAV starts with the visual target in the FoV of the camera. A circular trajectory is commanded to the UAV, which in turn keeps the visual target in the FoV and simultaneously avoids collision with the obstacle placed in the path. The $+x$ direction of the drone is represented by the red segment and the $+z$ direction of the camera is shown with a green arrow.

In this scenario, the UAV starts with its initial state set to $\left( {2}\,\hbox {m},-{2}\,\hbox {m},{1}\,\hbox {m},{225}^{\circ }\right)$ and with the visual target located at $\left( 0,0,0\right) \hbox {m}$. The commanded trajectory is a circle in *xy*-plane centered around the origin with a radius of ${2}\,\hbox {m}$. The robot starts with the visual target in view, but due to the circular trajectory, it has to continuously adjust its heading to keep up with the target. The soft constraint concerning the vertical location of the target in the virtual image plane allows the UAV to keep the commanded altitude even though the target is not at the center of the frame. Furthermore, an obstacle was added defined as $\pmb {\MakeLowercase {o}}_1 = \left( 0,2,2,1\right) \,\hbox {m}$. Due to its repulsion zone, the robot leaves its circular reference trajectory to avoid the obstacle. The 3D trajectory taken by the robot and the corresponding graphs are shown in Fig. 7.

#### Scenario 2 (perception, outside FoV)

**Figure 8:**
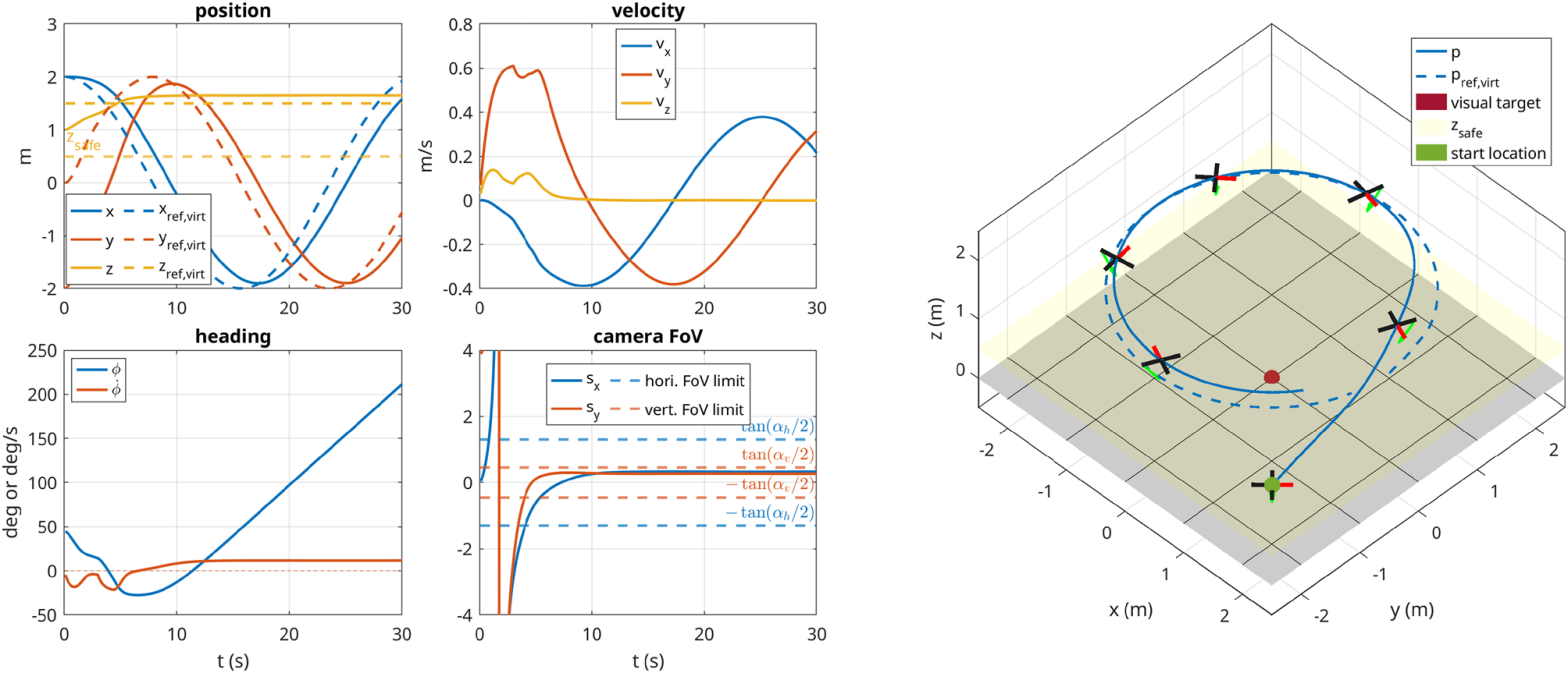
Scenario 2: The UAV starts with the visual target outside of the FoV of the camera and consequently has to perform almost a 180$^{\circ }$ turn to regain vision on the target, which represents a worst-case scenario to the optimizer.

This scenario is identical to scenario 1, except that the UAV starts with the camera pointing away from the target, i.e., with starting position $\left( {2}\,\hbox {m},-{2}\,\hbox {m},{1}\,\hbox {m},{45}^{\circ }\right)$, which represents a worst-case-scenario. Without the bias term in the perception cost, the optimization problem tends to converge in the sense that the camera keeps pointing away from the visual target. But with the formulation in (18), as shown in Fig. 8, the robot does manage to orient itself correctly to the target.

#### Scenario 3 (grasping approach)

**Figure 9:**
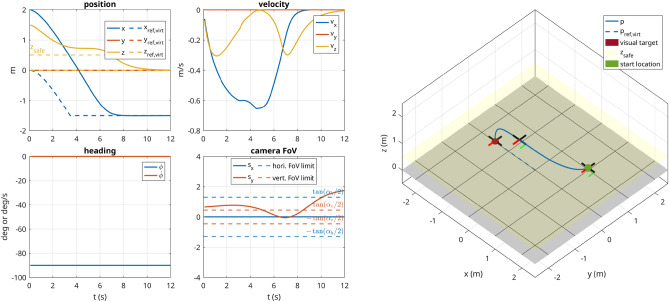
Scenario 3: The UAV approaches the target as if it would pick it up. It is not allowed to drop below 1 m until it is relatively close to the target in *xy*-plane and only then is the constraint lifted. At the same time, the perception constraint is faded out such that it does not over-constrain the problem.

In this scenario, the UAV starts with its initial state set to $\left( {2}\,\hbox {m},{0}\,\hbox {m},{2}\,\hbox {m},{-90}^{\circ }\right)$ and has the positional and visual target set to $\left( -{1.5}\,\hbox {m},{0}\,\hbox {m},{0}\,\hbox {m},{45}^{\circ }\right)$. Since the target is in the FoV, the MPC ignores the desired orientation and instead assures that the target stays in the FoV. A safety distance of 0.5 m was defined, which keeps the robot at a safe distance from the ground and only allows it to descend when close to the target (in *xy*-plane). At the same time, the perception constraint is dropped close to the target to avoid over-constraining the problem. The scenario is successfully executed as depicted in Fig. 9.

### Virtual experiment

#### Object detection pipeline

Visual servoing is an essential part of autonomous grasping as it guides the AM towards the payload using sensor feedback. By doing so, it relies heavily on the information provided by the visual sensors (e.g., cameras) as well as the processing chain used to extract various pieces of information such as position, orientation, size, or material.

For this application, the simulated UAV is equipped with the stereo depth camera *RealSense D435* that provides an RGB image with an associated depth channel (see Fig. 4). *Blob detection* is applied to the RGB image to detect objects based on their color and size. Using a pinhole camera model, the centroid of the detected blob is deprojected to a 3D point with the help of the depth information. Note that since the orientation of the payload is not required for the UG to operate, the vision pipeline can be kept lean and efficient, ideal for low-power embedded hardware.

#### Autonomous grasping in simulation

In this section, the components developed throughout this work are applied in a model scenario that has the disposal of a grasping object as its goal. The scenario is set up in Gazebo, a robotics simulator that performs a realistic simulation of the flight dynamics and sensors as well as the autopilot of a UAV inside a virtual environment.

**Figure 10:**
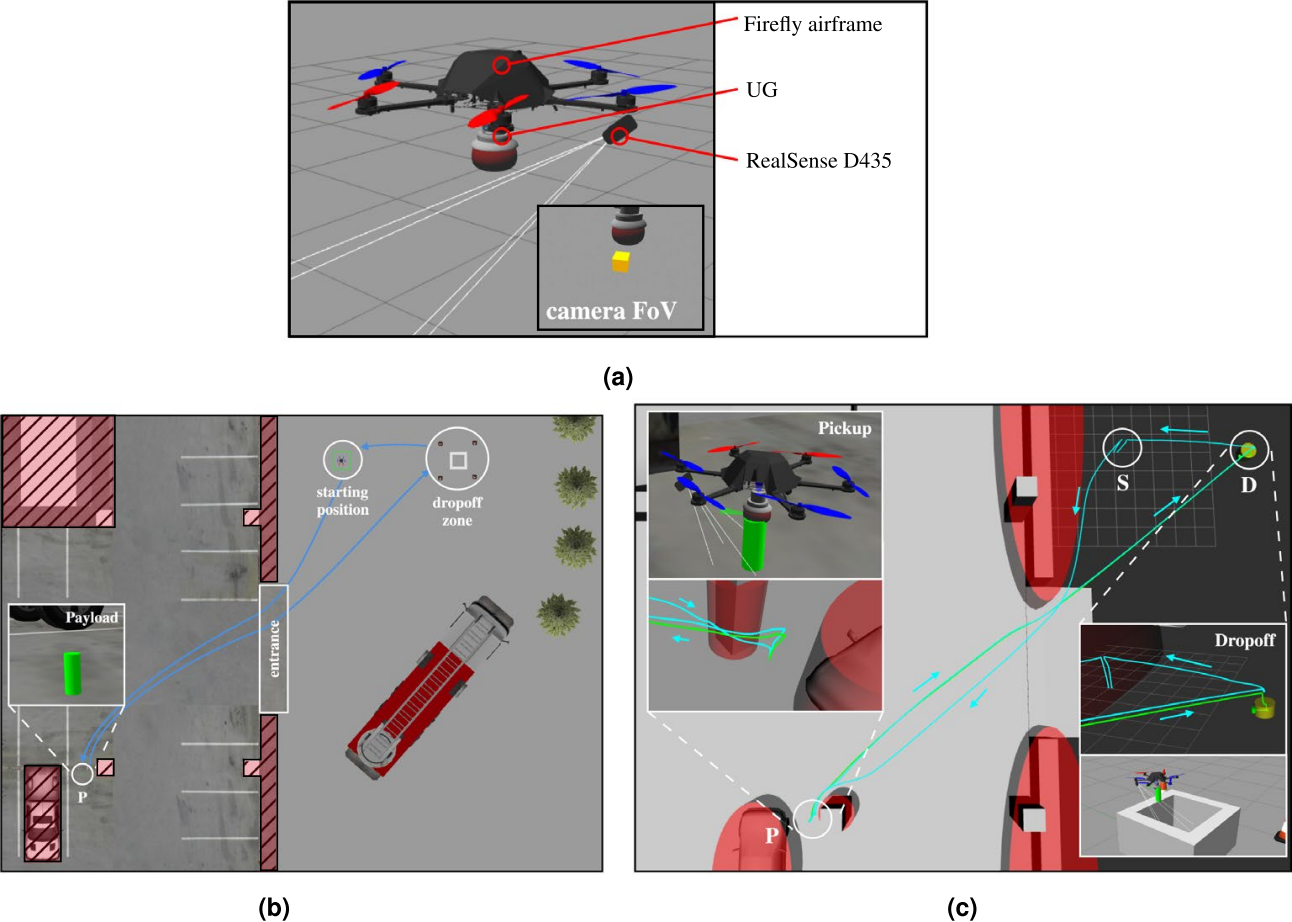
Virtual experiment; **(a)** The AM setup inside the Gazebo simulation consists of a hexacopter carrying the simulated UG and a stereo-vision camera (RealSense D435) for the localization of the payload in three dimensions; **(b)** The parking lot scenario in Gazebo. The payload was identified between the pickup truck and a pillar in a parking lot. The drone equipped with the UG is located at the starting position ’S’. It enters the parking via the entrance avoiding the walls and pillars. After establishing a successful grasp, the AM then transports the cylindrical object to the dropoff site; **(c)** The obstacles (walls, pillars, cars) are modeled as ellipses. The path taken by the UAV is traced in teal, and the path taken by the object is shown in green. The object is picked up and placed inside in the dropoff area ’D’ before the UAV returns and lands at the location ’S’.

The AM used throughout this work is depicted in Fig. 10a and consists of a hexacopter carrying the simulated UG as developed in the subsection on UAV modeling. It also features a stereo-vision camera that is used to localize the payload. A modified PX4^61^ serves as the autopilot for the drone and assumes communication with the UG via a custom interface module. The custom module further exposes the state information of the gripper to the outside world via MAVLink^62^ and consequently to ROS^63^ via a custom MAVROS plugin. Furthermore, the commands from ROS are forwarded to the gripper, making it fully integrated into the ROS ecosystem despite being directly connected to the Pixhawk autopilot.

The complete, system architecture is as depicted in Fig. 3. The mission planner pulls in all relevant information from ROS and provides the autopilot and gripper with the relevant commands based on the state of the mission. The intention is that the operator provides a rough description of the scenario (environment, obstacles, location of the payload), and then, based on that, the mission planner coordinates all the components to ensure the successful execution of the mission. The payload’s initial position can be a rough estimate as it gets updated automatically once it enters the FoV of the camera. In addition to the mission planner, a computer vision ROS node handles the object detection based on the color and shape (blob detection) and depth images and exposes the detected 3D coordinates of the payload to the mission planner.

To stay within the scope of this paper, the MPC is made aware of potential obstacles by manually specifying their bounds before launching the simulation. For real applications, online visual SLAM can be used to create a map of the environment in real-time, e.g.,^64^ and consequently generating and feeding a list of obstacles to the MPC. As mentioned in the cost function on tracking, the list of obstacles is sorted by their distance to the UAV (shortest distance to ellipsoid), and only the closest $N_o$ obstacles are fed into the MPC’s parameter vector (5).

#### Pick and place scenario

A grasping object has been identified in a parking garage close to a car. The goal of this simulation is to pick up the approximately localized object ($\pm {0.5}\,\hbox {m}$) and transport it to a dropoff site. The AM uses its onboard camera to further refine the localization of the object during the approach. The MPC handles the trajectory generation throughout the simulation and thus guides the UAV to the object respecting the FoV of the camera and vertical descent imposed by the gripper, while avoiding any obstacles such as the pillars, walls, and cars in the parking garage. Having reached the object, the control scheme is changed to force control via $\zeta$, where a nominal activation force of 2 N is tracked. After reaching steady-state on the exerted force, the simulated UG is closed, and the object is grasped successfully. The MPC now takes the AM to the dropoff site where the object is released. Finally, the AM returns to its starting position, where it lands again on the UG. The scenario is visualized in Fig. 10b.

#### Results

The path taken by the UAV is visualized in Fig. 10c alongside the obstacles known to the MPC. The *xy*-position and altitude are plotted separately in Fig. 13. The UAV successfully avoids all obstacles on its way and graciously approaches the target such that it can be grasped by the gripper. The robot then moves to the dropoff area, again avoiding all obstacles along its path. The MPC commands velocities (*x*, *y*, *z*, and yaw) at 100 Hz that are tracked via a set of cascading PID controllers for the roll, pitch, and yaw axis, resp., the velocities in *x* and *y*. The aforementioned SMC tracks the commanded velocity in *z*. The commanded and actual velocities are visualized in Fig. 13. It can be seen that the commanded velocity in *z*-direction approaches a very gentle − 0.06 $\text{ms}^{-1}$, which allows for a relatively smooth transition to the force-control scheme which is shown in detail in Fig. 12 between the 35 s and 43 s mark. A nominal force of 2 N is tracked by the SMC during the grasping interval. Once the activation force reaches steady-state, the gripper is closed at the 37 s mark. After having acquired a secure grasp, the UAV takes off (Fig. 13, 43 s mark). Consequently, the gripper’s load cell registers the weight of the payload (200 g), which is fed back to the SMC, which is thus aware of the changed mass of the system. A comprehensive video detailing the entire experiment, showcasing the UAV’s path, obstacle avoidance, approach to the object, and the grasping process, is available for a more nuanced understanding and visualization of the results (see Movie S1 in the Supplementary Materials for details).

**Figure 11:**
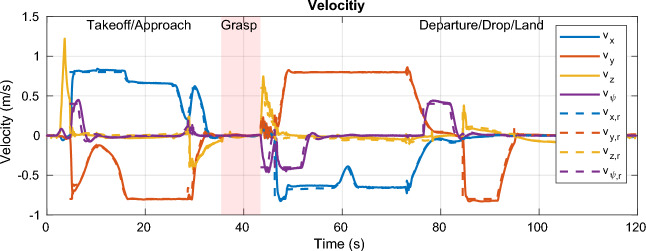
The MPC commands velocities in *x*, *y*, *z*, and yaw directions, which are tracked by the low-level controllers, including the SMC for altitude. The payload enters the field of view of the camera at the 30 s mark, where the MPC takes corrective actions such that the payload stays in view. The UAV carefully approaches the payload at the 37 s mark and departs towards the drop zone at the end of the grasping phase. After dropping the payload, the UAV lands back on the gripper at the start location.

**Figure 12:**
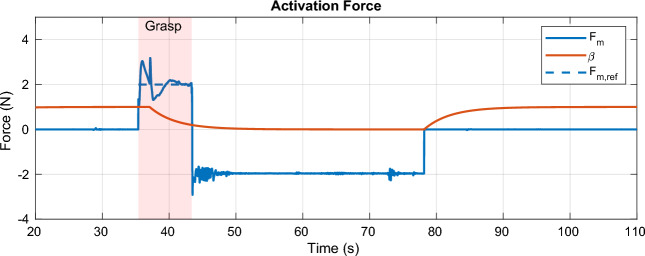
A nominal force of 2 N is tracked during the contact period between 35 s and 43 s. The gripper is closed after reaching steady-state at $t={37}\,\hbox {s}$. After takeoff, the force sensor is used to get an estimate for the weight of the payload, here 200 g, resp. − 1.95 N. At the 78 s mark, the payload is dropped in the drop-off area. The state variable $\beta$ represents the internal state of the gripper (1 for fluidized, 0 for stiff).

**Figure 13:**
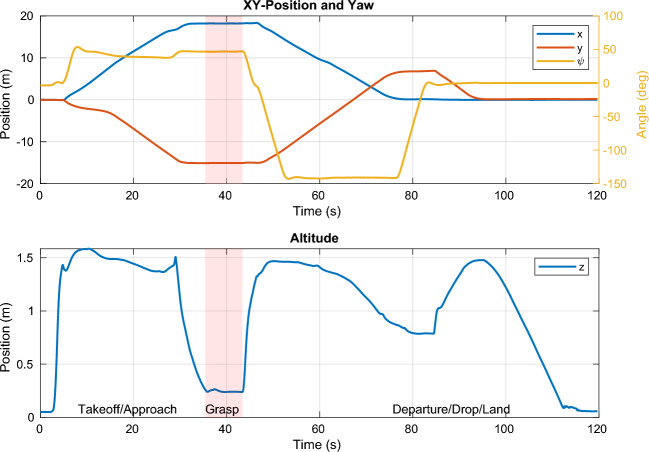
The *xy*-position and yaw angle (top) and the altitude (bottom). The visual constraints lead to the altitude to first rise before sharply decreasing as the UAV comes very close to the payload and the visual constraint is phased out. Likewise, a small adjustment in yaw can be observed at that point (29 s mark).

## Discussion

The primary findings of this study underscore the positive synergetic effects between mechanical intelligence, and state-of-the-art control techniques for AMs. Thanks to the passive intelligence built into the mechanics of the UG, the control system of the AM can be kept relatively straightforward, which is a net benefit over existing solutions that often require complicated and expensive algorithms to find the payload’s orientation to calculate the optimal angle of approach.

Leveraging the principles of granular jamming, the UG showcased adeptness in handling various objects, irrespective of their geometrical intricacies^34^. The trajectory optimization, when coupled with trajectory tracking, furnishes the UAV with the capability to achieve optimal flight paths in real-time. This combination of real-time trajectory optimization and force control paves the way for increased autonomy, enabling AMs to operate efficiently without constant human intervention.

The current research landscape, while burgeoning with innovations, often leans towards compensating shortcomings, e.g., pin-point accuracy requirements, by enhancing the UAV’s agility and precision. Although a viable approach, it adds substantial complexity and costs and is diametrically different from our proposed solution which shifts the complexity back to the mechanical system of the gripper where it is handled using the passive intelligence baked into the jamming membrane. Our approach offers an integrated solution that synergizes the strength of both the mechanical system as well as the control system to address the multifaceted challenges of aerial grasping.

This research shows promising avenues for a broad spectrum of sectors. With advancements in aerial grasping capabilities, industries demanding precise material handling in remote or elevated locations, such as construction or agriculture, stand to benefit immensely. The potential of the research is particularly striking in scenarios like disaster response, where the urgency to operate efficiently in challenging terrains is paramount. Moreover, the findings could catalyze a more extensive deployment of UAVs in diverse areas, including environmental monitoring and urban delivery tasks. On the design front, the insights from this study could guide the evolution of future UAV designs, optimizing them for more general aerial grasping tasks.

The UG’s design mainly favors grasping objects from the floor, which challenges alternate orientations like horizontal grasping as the granular material would accumulate unevenly around the object. Although this allows for simple grasping strategies, it does limit the use of the AM to cases with enough space for the drone’s body not to collide with the surroundings. We believe this issue can in part be addressed with design changes to the membrane and also on the control side, inspiration can be taken from various research articles dealing with building inspection requiring physical contact^65^.

## Conclusion

In our previous work, we developed a universal jamming gripper for UAVs, showing its grasping capabilities in real experiments. Although the grasping procedure requires neither precise positioning nor contact force control (major advantages of our design), the requirement for an operator in the loop was still detrimental to its reliability as controlling several parameters simultaneously quickly overburdened the pilots. This study thus marks the first step towards automated aerial grasping with UGs by taking the human out of the loop. Based on the experimental results of our previous work, a simulation model of a UG was developed upon which an adequate control strategy was built, consisting of a force tracker and an optimizing constraint trajectory generator. The control strategy was validated using numerical simulations and virtual experiments.

Future work consists of deploying and testing the developed solution on real hardware. Furthermore, the current blob detection-based vision pipeline is too simplistic to cope with real-world objects and we believe the system could benefit from modern learning-based methods. This could also enable the AM to detect suitable landing locations and thus capitalize on the AM’s ability to land or rest directly on the gripper also in unconventional places, e.g., on branches or posts. Prop wash is still likely to dislocate lightweight objects on approach although the trajectory taken by the AM reduces its effects. A solution to this could entice further mechanical changes to the AM, e.g., increasing the distance between the gripper and the UAV, or adding some form of shielding against the airflow. Moreover, global trajectory planning (pathfinding) is currently missing from our solution, limiting the AM’s operation space. Lastly, the AM could be a good candidate to include in a multi-robot cooperative aerial manipulation scheme for larger and heavier payloads, exploiting recent developments in inter-robot management schemes^66^ and combining those with the simplicity and unique abilities of our grasping solution.

## Supplementary Information

Supplementary Information.

Supplementary Information.

## Data Availability

The datasets used and/or analyzed during the current study are available from the corresponding author on reasonable request.
