# Supplementary material for: Constrained trajectory optimization and force control for UAVs with universal jamming grippers
Source: Sci Rep. 2024 May 25;14:11968. doi: 10.1038/s41598-024-62416-1 (PMC11636936; doi:10.1038/s41598-024-62416-1)
Supplement: Supplementary file 2 — Supplementary Information. [file 41598_2024_62416_MOESM2_ESM.docx]

**Supplementary Materials**

**Constrained Trajectory Optimization and Force Control for UAVs with Universal Jamming Grippers**

Paul Kremer, Hamed Rahimi Nohooji, and Holger Voos
hamed.rahimi@uni.lu

Supplementary Materials for this manuscript includes the following:

**Movie S1** (.mp4 format). Video of the experiment showing the automated grasping of a payload.
